# Free vibration of nanobeams with surface and dynamic flexoelectric effects

**DOI:** 10.1038/s41598-024-82002-9

**Published:** 2024-12-04

**Authors:** Peng Wang, JiaWei Xu, XiWen Zhang, YingHui Lv

**Affiliations:** School of Civil Engineering and Architecture, University of Jinan, Jinan, 250022 China

**Keywords:** Dynamic flexoelectricity, Surface effect, Flexoelectric effect, Piezoelectric nanobeam, Free vibration, Nanoscience and technology, Physics

## Abstract

In this paper, the free vibration of piezoelectric nanobeams considering static flexoelectric, dynamic flexoelectric, and surface effects is studied. Based on the theories of the Timoshenko beam and Euler-Bernoulli beam, a theoretical model of flexoelectric nanobeams is established and the governing equations and boundary conditions of this model are derived using the variational principle. Then, the analytical solution of the frequency equation is obtained by using the Navier method. Numerical results indicate that the size dependence of the dynamic flexoelectric effect is stronger than the surface effect. The surface and dynamic flexoelectric effects exhibit a competitive mechanism on the natural frequency.

## Introduction

Flexoelectricity is a special electromechanical coupling phenomenon that represents the electric polarization induced by non-uniform deformation in dielectric materials^1–3^. Flexoelectricity exists in all dielectric materials and becomes significant as structural size decreases due to size effects^4–6^. At the micro- and nano-scale, flexoelectricity is predicted to match or even dominate over piezoelectricity. With the development of nanotechnology, flexoelectricity has extensive application prospects and can be widely used in the design of micro- and nano- electronics devices, including sensors^7–9^, actuators^10,11^, and energy harvesters^12–14^.

In recent years, considerable progress has been made in the studies of the flexoelectric effect. In particular, the development of strain gradient theory has enhanced the accuracy of mechanical response descriptions^15–21^. For the static flexoelectric effect, static flexoelectric coefficients have been measured for various materials^22–24^ and fundamental models have been established^25–27^. The static bending and dynamic responses of fundamental mechanical structures such as beams^28–31^, plates^32–37^, and shells^38–41^ with static flexoelectric effect have been studied by many researchers. The dynamic flexoelectric effect has not attracted much attention from researchers until Kvasov et al.^42^ theoretically predicted the dynamic flexoelectric coefficients, the studies of dynamic flexoelectric effect have increased in recent years. Nguyen et al.^43^ analyzed the influence of dynamic flexoelectric effect on the free vibration of piezoelectric nanobeams. Deng et al.^44^ examined the influence of dynamic flexoelectric effect on the flexoelectric energy harvester. Wang et al.^45^ studied the free vibration of natural frequency for piezoelectric nanoplates with dynamic flexoelectric effect. Yu et al.^46^ addressed the dynamic flexoelectric effect on the bending and vibration of functionally graded piezoelectric nanobeams. Thai et al.^47^ developed a nonlinear dynamically consistent model with the impact of internal length, internal inertia gradient, and dynamic flexoelectric effect and analyzed the vibrational behaviors of the energy harvesters. All the above-mentioned references indicate that the influence of static and dynamic flexoelectricity on the static bending or dynamic responses of nanostructures is non-negligible. At the nanoscale, not only present the static and dynamic flexoelectric effects but also other size dependent phenomena, such as the surface effect. Experimental and theoretical studies showed that the surface effect also can significantly affect the mechanical behaviors of nanostructures^48–51^.

The surface effect refers to the phenomenon that the physical, chemical, and mechanical properties are distinct to macroscopic structures due to the increased surface-to-bulk ratio caused by the miniaturization of structures. To explore the influence of surface effect, Shen and Hu^27^ developed a new electro-enthalpy variation principle of a new surface energy density function including surface stress, surface piezoelectricity, and polarization of dielectric materials. Liang et al.^52,53^ investigated the bending and vibration behavior of Euler-Bernoulli nanobeams with surface and flexoelectric effects. Zhang et al.^54^ analyzed the electromechanical coupling of flexoelectric nanoplates with surface effect. Yue et al.^55^ examined the influence of flexoelectric and surface effects on the bending and vibration behaviors of Timoshenko piezoelectric nanobeams. Shingare et al.^56^ adopted Galerkin’s weighted residual and finite element (FE) methods to analyze the electromechanical behavior of Euler-Bernoulli nanobeams. Based on the Euler-Bernoulli theory, Gupta et al.^57^ studied the surface and flexoelectric effects on the electromechanical response of the Boron-Nitride reinforced nanocomposite beam under three different types of support. Zhou et al.^58^ addressed the surface effect on the electromechanical response of bilayer circular nanoplates. Presently, most studies on flexoelectric nanostructures with surface effects focus on the static flexoelectric effect, often neglecting dynamic flexoelectric effects. Recent research indicates that the dynamic flexoelectric effect significantly influences the vibration behavior of flexoelectric nanobeams, but the study of flexoelectric nanobeams considering the dynamic flexoelectric and surface effects has not been reported. How the dynamic flexoelectric effect affects the vibration behavior of flexoelectric nanobeams with consideration of surface effect is an interesting question to be discussed. This paper aims to study the free vibration of flexoelectric nanobeams by considering both dynamic flexoelectric and surface effects.

The present work examines the vibration response of the Timoshenko and Euler-Bernoulli piezoelectric nanobeams with static flexoelectric, dynamic flexoelectric, and surface effects. The governing equations and boundary conditions are deduced by using the variational principle in Sect. 2. Then, the analytical solution of frequency equations is derived using the Navier method in Sect. 3. The impacts of the static flexoelectric, dynamic flexoelectric, and surface effects on the natural frequencies of nanobeams are discussed in Sect. 4. Finally, the conclusions of this paper are presented in Sect. 5.

## Theoretical formulations

This paper aims to analyze the influence of static flexoelectric, dynamic flexoelectric, and surface effects on the vibration behavior of nanobeams. To describe the surface effect, we assume that the bulk and surface parts of the nanobeam are perfectly bonded, without any debonding and slipping. Therefore, we adopt classical elasticity theory for the bulk part and surface elasticity theory part for the surface part of the nanobeam, respectively. Based on this assumption, the internal energy density ${U_b}$ of the bulk part can generally be written as^27^

1 $${U_b}=\frac{1}{2}\left( {{\sigma _{ij}}{\varepsilon _{ij}}+{\sigma _{ijk}}{\varepsilon _{ij,k}}+{E_i}{P_i}} \right)$$

where ${\varepsilon _{ij}}$,${\sigma _{ij}}$,${\sigma _{ijk}}$ and ${\varepsilon _{ij,k}}$ are the components of the strain tensor, stress tensor, higher order stress tensor, and strain gradient tensor, respectively. ${E_i}$ and ${P_i}$ designate the elements of the electrical field tensor and the polarization tensor. ${\varepsilon _{ij}}$ and ${E_i}$ are defined as

2 $${\varepsilon _{ij}}=\frac{1}{2}({u_{i,j}}+{u_{j,i}}),{\text{ }}{E_i}= - {\varphi _{,i}}$$

where *u* and $\varphi$ denote the displacement vector and electric potential vector. Based on Eq. (1), the constitutive equations of the bulk part can be expressed as

3a $${\sigma _{ij}}={c_{ijkl}}{\varepsilon _{kl}}+{d_{ijk}}{P_k}$$

3b $${\sigma _{ijk}}={f_{ijkl}}{P_l}$$

3c $${E_i}={a_{ij}}{P_j}+{d_{jki}}{\varepsilon _{jk}}+{f_{jkli}}{\varepsilon _{jk,l}}$$

where ${c_{ijkl}}$, ${d_{ijk}}$, ${f_{ijkl}}$ and ${a_{ij}}$ are the elastic constant, the piezoelectric constant, the flexoelectric coupling coefficient, and the reciprocal dielectric susceptibility, respectively.

Regarding the surface portion, the surface energy density ${U_s}$ is given as follows:

4 $${U_s}={U_{s0}}+\frac{1}{2}\left( {\sigma _{{\alpha \beta }}^{s}\varepsilon _{{\alpha \beta }}^{s}+E_{\alpha }^{s}P_{\alpha }^{s}+{\Gamma _{\alpha \beta }}\varepsilon _{{\alpha \beta }}^{s}} \right)$$

where ${U_{s0}}$ is the material constant of the surface, *s* designates those related to the surface, and ${\Gamma _{\alpha \beta }}$ is surface residual stress which is usually taken as ${\sigma _0}{\delta _{\alpha \beta }}$, ${\delta _{\alpha \beta }}$ is the Kronecker delta. Similarly, the surface constitutive equations can be derived as^59^

5a $$\sigma _{{\alpha \beta }}^{s}={\sigma _0}{\delta _{\alpha \beta }}+c_{{\alpha \beta \gamma \delta }}^{s}\varepsilon _{{\gamma \delta }}^{s}+d_{{\alpha \beta \gamma }}^{s}P_{\gamma }^{s}$$

5b $$E_{\alpha }^{s}=a_{{\alpha \beta }}^{s}P_{\beta }^{s}+d_{{\beta \gamma \alpha }}^{s}\varepsilon _{{\beta \gamma }}^{s}$$

The expression for the total electric enthalpy density *H* can be written^60^

6 $$H={U_b}+{U_s} - \frac{1}{2}{\varepsilon _0}{\varphi _{,z}}{\varphi _{,z}}+{\varphi _{,z}}{P_z}$$

where ${\varepsilon _0}=8.85 \times {10^{ - 12}}{\text{C/V}} \cdot {\text{m}}$is the permittivity of a vacuum. In this work, dynamic flexoelectricity is considered, thus the density of kinetic energy can be described as^61^

7 $$K=\frac{1}{2}\rho {\dot {u}_i}{\dot {u}_i}+{M_{ij}}{\dot {u}_i}{\dot {P}_j}$$

where $\rho$ is the density and ${M_{ij}}$ is the flexodynamic tensor^42^. Subsequently, for a given work *W* done by external forces, the governing equations and boundary conditions can be derived from the variational principle, which takes the following form:

8 $$\delta \int_{0}^{T} { - \left( {\int_{\Omega } {Hd\Omega +K+W} } \right)} dt=0$$

### Modelling of Timoshenko piezoelectric nanobeam

**Fig. 1:**
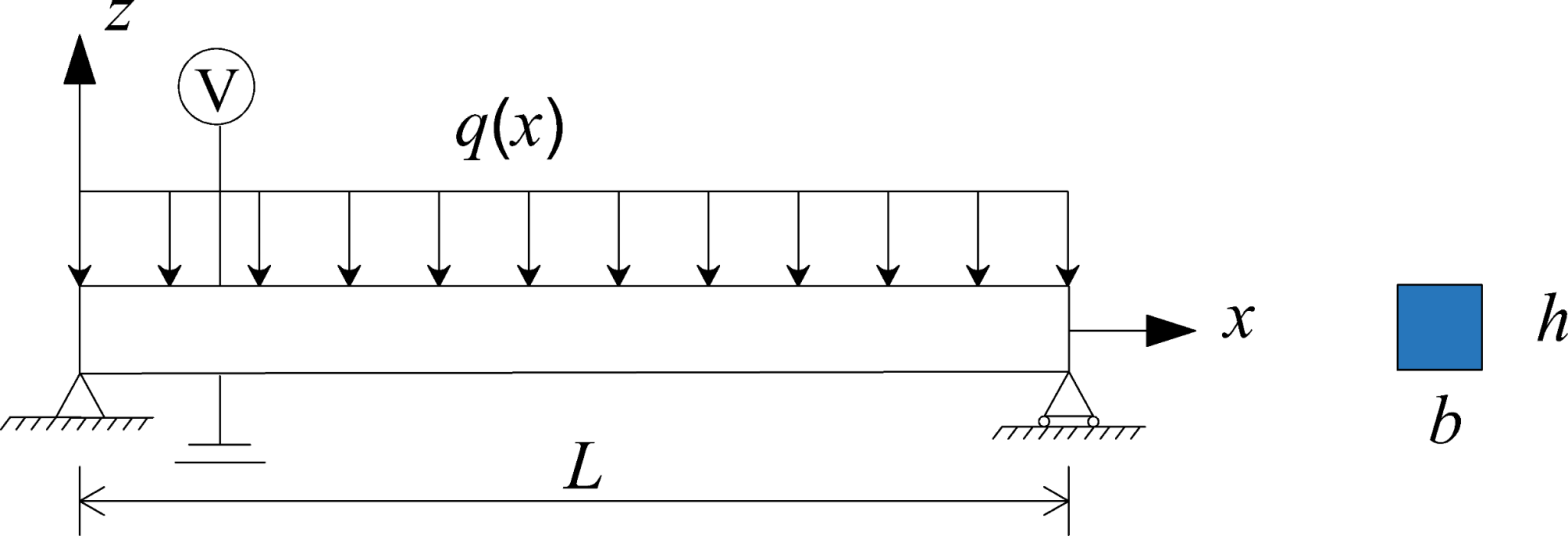
Schematic of a simply supported beam.

In this section, attention is focused on the vibration response of a simply supported piezoelectric nanobeam with the length *L*, thickness *h* and width *b* as shown in Fig. 1. Based on the Timoshenko beam theory and Euler-Bernoulli beam theory, a one-layer continuous beam model mechanical is established. This model consists of uniform and homogeneous materials. For the case of a Timoshenko beam without axial loads, the displacement field is assumed as

9 $${u_x}= - z\phi (x),{\text{ }}{u_z}=w(x,t)$$

where $\phi$ and *w* denote the rotation angle of the beam cross section and the transverse deflection of the beam, respectively. The non-zero strain and strain gradient components are derived from Eq. (2) as

10 $$\begin{gathered} {\text{ }}{\varepsilon _x}= - z\frac{{\partial \phi }}{{\partial x}},{\varepsilon _{x,z}}= - \frac{{\partial \phi }}{{\partial x}} \hfill \\ {\gamma _{xz}}=2{\varepsilon _{xz}}=( - \phi +\frac{{\partial w}}{{\partial x}}),{\gamma _{xz,x}}=( - \frac{{\partial \phi }}{{\partial x}}+\frac{{{\partial ^2}w}}{{\partial {x^2}}}) \hfill \\ \end{gathered}$$

For simplification of the analysis, the electric field is assumed to vary only along *z*-direction^27^, i.e.,

11 $${E_z}= - \frac{{\partial \varphi }}{{\partial z}}$$

Based on this simplification, the non-zero stresses, higher order stress and electric field of body portion can be expressed in the following form:

12a $${\sigma _x}={c_{11}}{\varepsilon _x}+{d_{31}}{P_z},{\text{ }}{\sigma _{xz}}=k{c_{44}}{\gamma _{xz}}$$

12b $${\sigma _{xxz}}={f_{3113}}{P_z},{\sigma _{xzx}}={f_{3131}}{P_z}$$

12c $${E_z}={a_{33}}{P_z}+{d_{31}}{\varepsilon _{xx}}+{f_{3113}}{\varepsilon _{xx,z}}+{f_{3131}}{\gamma _{xz,x}}$$

where $k=5/6$ is the shear correction factor for a rectangular cross section. Under the condition of zero free charge, Gauss’s law satisfies

13 $${\varepsilon _0}{E_{z,z}}+{P_{z,z}}=0$$

Substituting Eqs. (12c) and (13) into Eq. (11), and combining the electric boundary conditions: $\varphi (\frac{h}{2})=V,{\text{ }}\varphi ( - \frac{h}{2})=0$, we can express $\varphi$, ${P_z}$ and ${E_z}$ in the following form:

14a $$\varphi =\frac{{{d_{31}}}}{{\left( {1+{a_{33}}{\varepsilon _0}} \right)}}\left( {\frac{{{z^2}}}{2} - \frac{{{h^2}}}{8}} \right)\frac{{\partial \phi }}{{\partial x}}+\frac{V}{h}z+\frac{V}{2}$$

14b $${P_z}=\frac{{{\varepsilon _0}{d_{31}}}}{{\left( {1+{a_{33}}{\varepsilon _0}} \right)}}z\frac{{\partial \phi }}{{\partial x}}+\frac{{{f_{3113}}}}{{{a_{33}}}}\frac{{\partial \phi }}{{\partial x}}+\frac{{{f_{3131}}}}{{{a_{33}}}}(\frac{{\partial \phi }}{{\partial x}} - \frac{{{\partial ^2}w}}{{\partial {x^2}}}) - \frac{V}{{{a_{33}}h}}$$

14c $${E_z}= - \frac{{{d_{31}}}}{{\left( {1+{a_{33}}{\varepsilon _0}} \right)}}z\frac{{\partial \phi }}{{\partial x}} - \frac{V}{h}$$

Next, plugging Eq. (14b) into Eqs. (12a) and (12b), the stresses and higher order stresses can be rewritten as

15a $${\sigma _x}=\left( {\frac{{{\varepsilon _0}d_{{31}}^{2}}}{{1+{\varepsilon _0}{a_{33}}}} - {c_{11}}} \right)z\frac{{\partial \phi }}{{\partial x}}+\frac{{{d_{31}}{f_{3113}}}}{{{a_{33}}}}\frac{{\partial \phi }}{{\partial x}}+\frac{{{f_{3131}}{d_{31}}}}{{{a_{33}}}}\left( {\frac{{\partial \phi }}{{\partial x}} - \frac{{{\partial ^2}w}}{{\partial {x^2}}}} \right) - \frac{{{d_{31}}V}}{{{a_{33}}h}}$$

15b $${\sigma _{xz}}=k{c_{44}}\left( { - \phi +\frac{{\partial w}}{{\partial x}}} \right)$$

15c $${\sigma _{xxz}}=\frac{{{\varepsilon _0}{d_{31}}{f_{3113}}}}{{1+{\varepsilon _0}{a_{33}}}}z\frac{{\partial \phi }}{{\partial x}}+\frac{{f_{{3113}}^{2}}}{{{a_{33}}}}\frac{{\partial \phi }}{{\partial x}}+\frac{{{f_{3131}}{f_{3113}}}}{{{a_{33}}}}\left( {\frac{{\partial \phi }}{{\partial x}} - \frac{{{\partial ^2}w}}{{\partial {x^2}}}} \right) - \frac{{{f_{3113}}V}}{{{a_{33}}h}}$$

15d $$\begin{array}{*{20}{c}} {{\sigma _{xzx}}=\frac{{{\varepsilon _0}{d_{31}}{f_{3131}}}}{{1+{\varepsilon _0}{a_{33}}}}z\frac{{\partial \phi }}{{\partial x}}+\frac{{{f_{3131}}{f_{3113}}}}{{{a_{33}}}}\frac{{\partial \phi }}{{\partial x}}+\frac{{f_{{3131}}^{2}}}{{{a_{33}}}}\left( {\frac{{\partial \phi }}{{\partial x}} - \frac{{{\partial ^2}w}}{{\partial {x^2}}}} \right) - \frac{{{f_{3131}}V}}{{{a_{33}}h}}} \end{array}$$

Simultaneously, the surface stress $\sigma _{x}^{s}$ is derived from Eq. (5) as

16 $$\sigma _{x}^{s}={\sigma _0}+\left( {\frac{{{\varepsilon _0}{d_{31}}d_{{31}}^{s}}}{{1+{\varepsilon _0}{a_{33}}}} - c_{{11}}^{s}} \right)z\frac{{\partial \phi }}{{\partial x}}+\frac{{d_{{31}}^{s}{f_{3113}}}}{{{a_{33}}}}\frac{{\partial \phi }}{{\partial x}}+\frac{{{f_{3131}}d_{{31}}^{s}}}{{{a_{33}}}}\left( {\frac{{\partial \phi }}{{\partial x}} - \frac{{{\partial ^2}w}}{{\partial {x^2}}}} \right) - \frac{{d_{{31}}^{s}V}}{{{a_{33}}h}}$$

Then, the first variation of the total electric enthalpy can be expressed as

17 $$\begin{gathered} \delta \mathop \smallint \nolimits_{0}^{T} \mathop \smallint \nolimits_{\text{ }\!\!\Omega\!\!\text{ }}Hd\text{ }\!\!\Omega\!\!\text{ }=\mathop \smallint \nolimits_{0}^{T} \mathop \smallint \nolimits_{V} \left( {{\sigma _x}\delta {\varepsilon _x}+{\sigma _{xz}}\delta {\gamma _{xz}}+{\sigma _{xxz}}\delta {\varepsilon _{x,z}}+{\sigma _{xzx}}\delta {\gamma _{xz,x}}+{E_z}\delta {P_z}} \right)dVdt \hfill \\ {\text{ }}+\mathop \smallint \nolimits_{0}^{T} \mathop \smallint \nolimits_{s} \sigma _{x}^{s}\delta \varepsilon _{x}^{s}dSdt+\mathop \smallint \nolimits_{0}^{T} \mathop \smallint \nolimits_{V} \left( { - {\varepsilon _0}{\varphi _{,z}}\delta {\varphi _{,z}}+{\varphi _{,z}}\delta {P_z}+{P_3}\delta {\varphi _{,z}}} \right)dVdt \hfill \\ {\text{ =}}\mathop \smallint \nolimits_{0}^{T} \mathop \smallint \nolimits_{0}^{L} \left[ { - \left( {Q - \frac{{\partial {M^\gamma }}}{{\partial x}} - \frac{{\partial \left( {{M^s}+M} \right)}}{{\partial x}}} \right)\delta \phi - \mathop \smallint \nolimits_{0}^{L} \left( {{Q^s}+\frac{{\partial Q}}{{\partial x}} - \frac{{{\partial ^2}{M^\gamma }}}{{\partial {x^2}}}} \right)\delta w} \right]{\text{d}}xdt \hfill \\ {\text{ }}+\mathop \smallint \nolimits_{0}^{T} [(Q - \frac{{\partial {M^\gamma }}}{{\partial x}})\delta w - (M+{M^\gamma })\delta \phi +{M^\gamma }\delta {w_{,x}}]|_{{x=0}}^{{x=L}}dt{\text{ }} \hfill \\ \end{gathered}$$

where

18 $$\begin{gathered} Q=\int_{A} {{\sigma _{xz}}} dA,{\text{ }}M=\int_{A} {({\sigma _x}z+{\sigma _{xxz}})} dA,{\text{ }}{M^\gamma }=\int_{A} {{\sigma _{xzx}}dA,} \hfill \\ {M^s}=\int_{C} {\sigma _{x}^{s}} zdC,{\text{ }}{Q^s}=b\kappa ({\left. {\sigma _{x}^{s}} \right|_{h/2}} - {\left. {\sigma _{x}^{s}} \right|_{ - h/2}}) \hfill \\ \end{gathered}$$

are the shear forces and resultant bending moments of the beam, *A* is the beam cross section, *C* is the perimeter of the beam cross section and $\kappa$ is the curvature of the bending element approximated to$\frac{{{\partial ^2}w}}{{\partial {x^2}}}$.

The kinetic energy of the beam can be written as

19 $$K=\int_{V} {\frac{1}{2}} \rho \left[ {{{\left( {\frac{{\partial w}}{{\partial t}}} \right)}^2}+{{\left( {\frac{{\partial \phi }}{{\partial t}}} \right)}^2}} \right]dV+\int_{V} {{M_{33}}} \dot {w}{\dot {P}_z}dV$$

From Eq. (14b), the variation of time derivative of the polarization ${P_z}$ can be obtained as

20 $$\delta {\dot {P}_z}=\frac{{\partial {{\dot {P}}_z}}}{{\partial {{\dot {\phi }}_{,x}}}}\delta {\dot {\phi }_{,x}}+\frac{{\partial {{\dot {P}}_z}}}{{\partial {{\dot {w}}_{,xx}}}}\delta {\dot {w}_{,xx}}$$

Thus, the first variation of the kinetic energy can be derived as

21 $$\delta \int_{0}^{T} K dt=\int_{0}^{T} {\left\{ {\int_{0}^{L} {(\rho A} \dot {w}\delta \dot {w}+\rho I\dot {\phi }\delta \dot {\phi })dx+\int_{0}^{L} {\int_{A} {{M_{33}}\dot {w}\delta {{\dot {P}}_z}+{M_{33}}A{{\dot {P}}_z}\delta \dot {w}dAdx} } } \right\}} dt$$

The virtual work done by the external forces takes:

22 $$\delta W=\int_{0}^{L} {q\delta wdx}$$

Substituting Eqs. (17), (21) and (22) into Eq. (8), and taking advantage of the arbitrariness of $\delta w$ and $\delta \phi$, we can obtain the governing equations as follows:

23a $$Q - \frac{{\partial {M^\gamma }}}{{\partial x}} - \frac{{\partial \left( {{M^s}+M} \right)}}{{\partial x}} - \rho I\frac{{{\partial ^2}\phi }}{{\partial {t^2}}}+\int_{A} {{M_{33}}\frac{{{\partial ^2}}}{{\partial x\partial t}}} \left( {\dot {w}\frac{{\partial {{\dot {P}}_z}}}{{\partial {{\dot {\phi }}_{,x}}}}} \right)dA=0$$

23b $${Q_s}+\frac{{\partial Q}}{{\partial x}} - \frac{{{\partial ^2}{M^\gamma }}}{{\partial {x^2}}} - \rho A\frac{{{\partial ^2}w}}{{\partial {t^2}}} - \int_{A} {{M_{33}}{{\ddot {P}}_z}} dA - \int_{A} {{M_{33}}\frac{{{\partial ^3}}}{{\partial {x^2}\partial t}}} \left( {\dot {w}\frac{{\partial {{\dot {P}}_z}}}{{\partial {{\dot {w}}_{,xx}}}}} \right)dA+q=0$$

with the corresponding boundary conditions

24 $$\begin{gathered} Q - \frac{{\partial {M^\gamma }}}{{\partial x}}+\int_{A} {{M_{33}}\frac{{{\partial ^2}}}{{\partial x\partial t}}} \left( {\dot {w}\frac{{\partial {{\dot {P}}_z}}}{{\partial {{\dot {w}}_{,xx}}}}} \right)dA{\text{ or }}w \hfill \\ M+{M^\gamma } - \int_{A} {{M_{33}}} \frac{\partial }{{\partial t\partial x}}\left( {\dot {w}\frac{{\partial \dot {P}}}{{\partial {{\dot {\phi }}_{,x}}}}} \right)dA{\text{ or }}\phi \hfill \\ - {M^\gamma } - \int_{A} {{M_{33}}} \frac{\partial }{{\partial t\partial x}}\left( {\dot {w}\frac{{\partial \dot {P}}}{{\partial {{\dot {w}}_{,xx}}}}} \right)dA{\text{ or }}{w_{,x}} \hfill \\ \end{gathered}$$

are prescribed at $x=0$ and $x=L$. Combining Eqs. (15) and (18), the bending moments and shear force are given by the following expression

25 $$\begin{gathered} \begin{array}{*{20}{c}} {M=\left( {\frac{{{\varepsilon _0}d_{{31}}^{2}}}{{1+{\varepsilon _0}{a_{33}}}} - {c_{11}}} \right)I\frac{{\partial \phi }}{{\partial x}}+\left[ {\frac{{f_{{3113}}^{2}}}{{{a_{33}}}}\frac{{\partial \phi }}{{\partial x}}+\frac{{{f_{3131}}{f_{3113}}}}{{{a_{33}}}}\left( {\frac{{\partial \phi }}{{\partial x}} - \frac{{{\partial ^2}w}}{{\partial {x^2}}}} \right) - \frac{{{f_{3113}}V}}{{{a_{33}}h}}} \right]A} \end{array}, \hfill \\ \begin{array}{*{20}{c}} {Q=k{c_{44}}A\left( { - \phi +\frac{{\partial w}}{{\partial x}}} \right),{M^\gamma }=\left[ {\frac{{{f_{3131}}{f_{3113}}}}{{{a_{33}}}}\frac{{\partial \phi }}{{\partial x}}+\frac{{f_{{3131}}^{2}}}{{{a_{33}}}}\left( {\frac{{\partial \phi }}{{\partial x}} - \frac{{{\partial ^2}w}}{{\partial {x^2}}}} \right) - \frac{{{f_{3131}}V}}{{{a_{33}}h}}} \right]A,} \\ {{M^s}=\left( {\frac{{{\varepsilon _0}{d_{31}}d_{{31}}^{s}}}{{1+{\varepsilon _0}{a_{33}}}} - c_{{11}}^{s}} \right){I_s}\frac{{\partial \phi }}{{\partial x}},{\text{ }}{Q^s}=2b{\sigma _0}\frac{{{\partial ^2}w}}{{{\text{~}}\partial {x^2}}}} \end{array} \hfill \\ \end{gathered}$$

where $I=\frac{{b{h^3}}}{{12}}$and ${I_s}=\frac{{{h^3}}}{6}+\frac{{b{h^2}}}{2}$ for rectangular cross section.

Then, plugging Eqs. (14b) and (25) into Eq. (23), we rearrange the governing equations into the following form:

26 $$\begin{gathered} {a_1}\phi +{a_3}\frac{{{\partial ^2}\phi }}{{\partial {x^2}}} - {a_1}\frac{{\partial w}}{{\partial x}} - {a_2}\frac{{{\partial ^3}w}}{{\partial {x^3}}} - {a_4}\frac{{{\partial ^2}}}{{\partial {t^2}}}(\frac{{\partial w}}{{\partial x}})= - \rho I\frac{{{\partial ^2}\phi }}{{\partial {t^2}}} \hfill \\ {a_1}\frac{{\partial \phi }}{{\partial x}}+{a_2}\frac{{{\partial ^3}\phi }}{{\partial {x^3}}} - {b_1}\frac{{{\partial ^2}w}}{{\partial {x^2}}} - {b_2}\frac{{{\partial ^4}w}}{{\partial {x^4}}}+{a_4}\frac{{{\partial ^2}}}{{\partial {t^2}}}(\frac{{\partial w}}{{\partial x}}) - {a_5}\frac{{{\partial ^2}}}{{\partial {t^2}}}(\frac{{{\partial ^2}\phi }}{{\partial {x^2}}})=q - \rho A\frac{{{\partial ^2}w}}{{\partial {t^2}}} \hfill \\ \end{gathered}$$

where

27 $$\begin{gathered} {a_1}=k{c_{44}}A,{a_2}=\left( {\frac{{{f_{3131}}{f_{3113}}}}{{{a_{33}}}}+\frac{{f_{{3131}}^{2}}}{{{a_{33}}}}} \right)A,{b_1}=2b{\sigma _0}+{a_1},{b_2}=\frac{{f_{{3131}}^{2}}}{{{a_{33}}}}A, \hfill \\ {a_3}=\left( {\frac{{{\varepsilon _0}d_{{31}}^{2}}}{{1+{\varepsilon _0}{a_{33}}}} - {c_{11}}} \right)I+\left( {\frac{{{\varepsilon _0}{d_{31}}d_{{31}}^{s}}}{{1+{\varepsilon _0}{a_{33}}}} - c_{{11}}^{s}} \right){I_s}+\left( {\frac{{f_{{3113}}^{2}}}{{{a_{33}}}}+2\frac{{{f_{3131}}{f_{3113}}}}{{{a_{33}}}}+\frac{{f_{{3131}}^{2}}}{{{a_{33}}}}} \right)A, \hfill \\ {a_4}=\frac{{{M_{33}}A\left( {{f_{3113}}+{f_{3131}}} \right)}}{{{a_{33}}}},{a_5}=\frac{{2{M_{33}}A{f_{3131}}}}{{{a_{33}}}}{\text{.}} \hfill \\ \end{gathered}$$

### Modelling of Euler-Bernoulli piezoelectric nanobeam

For the aspect ratio $L/h \geqslant 20$ of slender beams, the Euler-Bernoulli beam theory can be adopted. Accordingly, we assume $\phi ={w_{,x}}$ and ignore the rotary inertia in the kinetic energy. Based on these simplifications, the governing equation for the Euler-Bernoulli beam can be obtained using the same method as in the previous Sect. 

28 $$\frac{{{\partial ^2}M}}{{\partial {x^2}}}+\frac{{{\partial ^2}{M^s}}}{{\partial {x^2}}} - \rho A\ddot {w} - \int_{A} {{M_{33}}{{\ddot {P}}_z}} dA - \int_{A} {{M_{33}}\frac{{{\partial ^3}}}{{\partial {x^2}\partial t}}} \left( {\dot {w}\frac{{\partial {{\dot {P}}_z}}}{{\partial {{\dot {w}}_{,xx}}}}} \right)dA+q+{Q_s}=0$$

Simultaneously, the boundary conditions can be determined as

29 $$\begin{gathered} - \frac{{\partial M}}{{\partial x}} - \frac{{{\partial ^2}{M^s}}}{{\partial {x^2}}}+\int_{A} {{M_{33}}\frac{{{\partial ^2}}}{{\partial x\partial t}}} \left( {\dot {w}\frac{{\partial {{\dot {P}}_z}}}{{\partial {{\dot {w}}_{,xx}}}}} \right)dA{\text{ or }}w \hfill \\ M - \int_{A} {{M_{33}}\frac{\partial }{{\partial t}}} \left( {\dot {w}\frac{{\partial {{\dot {P}}_z}}}{{\partial {{\dot {w}}_{,xx}}}}} \right)dA{\text{ or }}{w_{,x}} \hfill \\ \end{gathered}$$

are prescribed on $x=0$ and $x=L$. The bending moment and the surface bending moment in Eq. (29) are expressed as

30 $$\begin{gathered} \begin{array}{*{20}{c}} {M=\left( {\frac{{{\varepsilon _0}d_{{31}}^{2}}}{{1+{\varepsilon _0}{a_{33}}}} - {c_{11}}} \right)I\frac{{{\partial ^2}w}}{{\partial {x^2}}}+A\left[ {\frac{{f_{{3113}}^{2}}}{{{a_{33}}}}\frac{{{\partial ^2}w}}{{\partial {x^2}}} - \frac{{{f_{3113}}V}}{{{a_{33}}h}}} \right]} \end{array} \hfill \\ {M_s}=\left( {\frac{{{\varepsilon _0}{d_{31}}d_{{31}}^{s}}}{{1+{\varepsilon _0}{a_{33}}}} - c_{{11}}^{s}} \right){I_s}\frac{{{\partial ^2}w}}{{\partial {x^2}}} \hfill \\ \end{gathered}$$

As a result, the governing Eq. (28) for the rectangular cross section can be re-expressed in the following form:

31 $$\begin{gathered} \left[ {\frac{{b{h^3}}}{{12}}{c_{11}} - \frac{{b{h^3}{\varepsilon _0}d_{{31}}^{2}}}{{1+{\varepsilon _0}{a_{33}}}} - \frac{{Af_{{3113}}^{2}}}{{{a_{33}}}}+c_{{11}}^{s}\left( {\frac{{{h^3}}}{6}+\frac{{b{h^2}}}{2}} \right) - \frac{{{\varepsilon _0}{d_{31}}d_{{31}}^{s}}}{{1+{\varepsilon _0}{a_{33}}}}} \right]\frac{{{\partial ^4}w}}{{\partial {x^4}}}+\rho A\frac{{{\partial ^2}w}}{{\partial {t^2}}} \hfill \\ +\left( {\frac{{2{M_{33}}Af_{{3113}}^{2}}}{{{a_{33}}}}\frac{{{\partial ^4}w}}{{\partial {x^2}\partial {t^2}}}} \right)=q+2b{\sigma _0}\frac{{{\partial ^2}w}}{{\partial {x^2}}} \hfill \\ \end{gathered}$$

## Free vibration

In this section, the static flexoelectric effect, dynamic flexoelectric effect and surface effect on the natural frequency of a free vibration of simply supported piezoelectric nanobeam is studied. For the Timoshenko beam model, the distributed load *q* is set to zero. By using Navier’s method, the transverse displacement and rotation can be assumed as follows:

32 $$w(x,t)=\sum\limits_{{n=1}}^{\infty } {{W_n}\sin \frac{{n\pi x}}{L}{e^{i{\omega _n}t}}} ,{\text{ }}\phi (x,t)=\sum\limits_{{n=1}}^{\infty } {{\Phi _n}\cos \frac{{n\pi x}}{L}{e^{i{\omega _n}t}}}$$

where ${\omega _n}$ is the natural frequency, ${W_n}$ and ${\Phi _n}$ represent the amplitude of translation and rotation, respectively. Substituting Eq. (32) into Eq. (26), the final governing equation for the free vibration of the Timoshenko nanobeam can be described as

33a $$\left[ {{a_1} - {a_3}{{\left( {\frac{{n\pi }}{L}} \right)}^2} - \rho I\omega _{n}^{2}} \right]\text{ }{{\Phi }_{n}}+\left[ { - {a_1}\frac{{n\pi }}{L}+{a_2}{{\left( {\frac{{n\pi }}{L}} \right)}^3}+\frac{{n\pi }}{L}\omega _{n}^{2}{a_4}} \right]{W_n}=0,$$

33b $$\begin{gathered} \left[ { - {a_1}\frac{{n\pi }}{L}+{a_2}{{\left( {\frac{{n\pi }}{L}} \right)}^3}+\frac{{n\pi }}{L}\omega _{n}^{2}{a_4}} \right]\text{ }{{\Phi }_{n}} \\     +\left[ {{b_1}{{\left( {\frac{{n\pi }}{L}} \right)}^2} - {b_2}{{\left( {\frac{{n\pi }}{L}} \right)}^4} - {{\left( {\frac{{n\pi }}{L}} \right)}^2}\omega _{n}^{2}{a_5} - \rho A\omega _{n}^{2}} \right]{W_n}=0 \hfill \\ \end{gathered}$$

From Eq. (33a), in order to have the non-trivial solutions of ${\Phi _n}$ and ${W_n}$, the frequency equation can be simplified as

34 $${c_1}\omega _{n}^{4} - {c_2}\omega _{n}^{2}+{c_3}=0$$

where

35 $$\begin{gathered} {c_1}={\rho ^2}AI+{\left( {\frac{{n\pi }}{L}} \right)^2}\left( { - a_{4}^{2}+\rho I{a_5}} \right), \hfill \\ {c_2}=\rho A{a_1}+\left( {\rho I{b_1}+{a_1}{a_5} - 2{a_1}{a_4} - \rho A{a_3}} \right){\left( {\frac{{n\pi }}{L}} \right)^2}+\left( { - \rho I{b_2}+2{a_2}{a_4} - {a_3}{a_5}} \right){\left( {\frac{{n\pi }}{L}} \right)^4} \hfill \\ {c_3}=\left( { - {a_3}{b_1}+{a_1}{b_2} - 2{a_1}{a_2}} \right){\left( {\frac{{n\pi }}{L}} \right)^4}+\left( {a_{2}^{2} - {a_3}{b_2}} \right){\left( {\frac{{n\pi }}{L}} \right)^6} \hfill \\ \end{gathered}$$

From Eq. (34), the expression of $\omega _{n}^{2}$ can be obtained as

36 $$\omega _{n}^{2}=\frac{{{c_2} - \sqrt {c_{2}^{2} - 4{c_1}{c_3}} }}{{2{c_1}}}$$

and the real positive root of ${\omega _n}$ is the natural frequency.

Meanwhile, a similar method can also be applied to the Euler-Bernoulli beam model. The natural frequency can be expressed in the following form:

37 $$\omega _{n} = \frac{{\frac{{n\pi }}{L}\sqrt {\left[ {\frac{{bh^{3} }}{{12}}c_{{11}} - \frac{{bh^{3} d_{{31}}^{2} \varepsilon _{0} }}{{a_{{33}} }} - \frac{{bhf_{{3113}}^{2} }}{{a_{{33}} }} + c_{{11}}^{s} \left( {\frac{{h^{3} }}{6} + \frac{{bh^{2} }}{2}} \right) - \frac{{bh^{3} d_{{31}} d_{{31}}^{s} \varepsilon _{0} }}{{a_{{33}} }}} \right]\left( {\frac{{n\pi }}{L}} \right)^{2} + 2b\sigma _{0} } }}{{\sqrt {\rho A - \frac{{2Af_{{3113}} M_{{33}} }}{{a_{{33}} }}} \left( {\frac{{n\pi }}{L}} \right)^{2} }}$$

## Numerical results and discussions

In numerical computations, the strontium titanate (STO) is chosen as a sample material for case study, the bulk and surface material properties at room temperature are given as^42,62,63^$${c_{11}}=371{\text{GPa}}$$, $${c_{44}}=132{\text{GPa}}$$, $$\rho =5120{\text{kg}}/{{\text{m}}^3}$$, $$c_{{11}}^{s}=83.1{\text{N}}/{\text{m}}$$,$${\sigma _0}=1{\text{N}}/{\text{m}}$$,$${f_{3113}}= - 1.3{\text{V}}$$$${f_{3131}}= - 0.28{\text{V}}$$, $${a_{33}}=0.47 \times {10^8}{\text{Vm}}/{\text{C}}$$. Although Kvasov et al.^42^ have reported the dynamic flexoelectric coefficient of the strontium titanate (STO), in this manuscript, we choose the range of dynamic flexoelectric coefficient from $$1 \times {10^{ - 5}}{\text{V}}{{\text{s}}^{\text{2}}}{{\text{m}}^{{\text{-2}}}} - 5 \times {10^{ - 8}}{\text{V}}{{\text{s}}^{\text{2}}}{{\text{m}}^{{\text{-2}}}}$$ to observe the obvious variation in the natural frequency. Additionally, we set the beam width is equal to the beam height (i.e., $$b=h$$) and the applied voltage $$V=0$$ in this paper. To better show the results, the following normalized natural frequency $$\omega _{n}^{{{\text{norm}}}}={\omega _n}/\omega _{n}^{{{\text{elastic}}}}$$ is calculated, in which $$\omega _{n}^{{{\text{elastic}}}}$$ is the natural frequency of corresponding classical elastic beam models (i.e., Timoshenko and Euler-Bernoulli beams without piezoelectric and flexoelectric effects).

### Effect of the dynamic flexoelectric effect

**Fig. 2:**
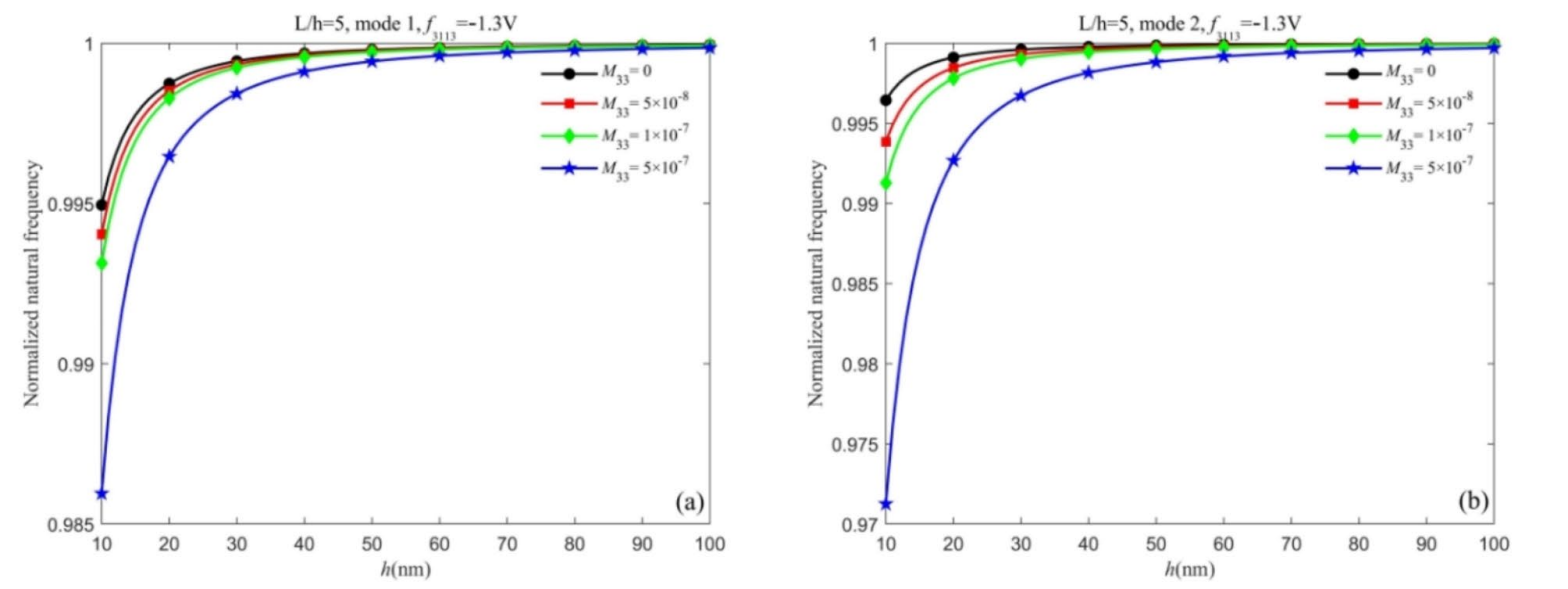
Influence of dynamic flexoelectric effect on the normalized natural frequency of Timoshenko beam (a) the first vibration mode and (b) the second vibration mode.

**Fig. 3:**
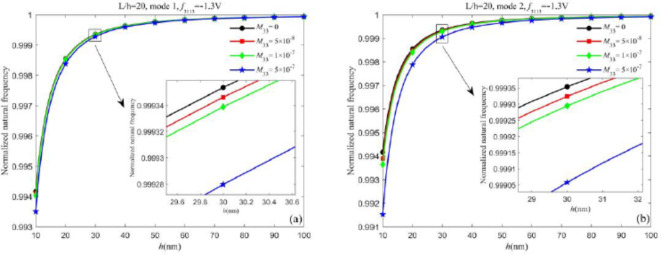
Influence of dynamic flexoelectric effect on the normalized natural frequency of Euler-Bernoulli beam (a) the first vibration mode and (b) the second vibration mode.

First, we analyze the dynamic flexoelectric effect on the natural frequencies. Figures 2 and 3 show the influence of dynamic flexoelectricity on the normalized natural frequency of different vibration modes for both nanobeams when neglecting the surface effect. When the dynamic flexoelectric effect is considered, the kinetic energy density is added and the equivalent mass also increases, therefore the natural frequency is decreased. From Figs. 2 and 3, the dynamic flexoelectric effect becomes prominent with the decrease of the thickness. When the beam becomes thicker, the influence of dynamic flexoelectricity on natural frequencies becomes weak until negligible and the normalized natural frequency is closer to 1. It demonstrates that dynamic flexoelectricity is size-dependent. For the first vibration mode of both nanobeams, it can be observed that the variation of the normalized natural frequency under different dynamic flexoelectric coefficients is minimal and can be neglected. However, for higher vibration modes, the normalized natural frequencies of both types of nanobeams decrease as the dynamic flexoelectric coefficient increases. These results clearly indicate that the dynamic flexoelectric effect is more influential for higher vibration modes. Thus, dynamic flexoelectricity is an important factor that needs to be considered in the analysis of piezoelectric nanobeams for higher vibration modes. It is worth mentioning that the role of the dynamic flexoelectric effect is more pronounced in the Timoshenko beam model compared to the Euler-Bernoulli beam model.

**Fig. 4:**
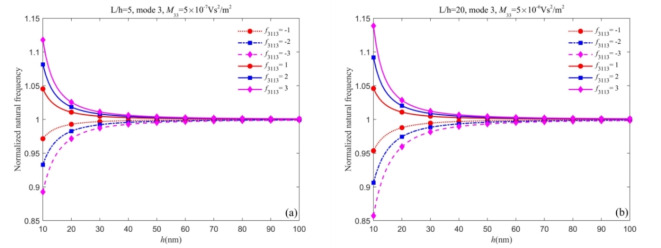
Variation of normalized natural frequency versus static flexoelectric coefficients (a)Timoshenko beam and (b) Euler-Bernoulli beam.

On the other hand, the choice of positive and negative static flexoelectric coefficients also affects the natural frequencies. Figure 4 illustrates the variation in the normalized natural frequency of both nanobeams with thickness *h* for different static flexoelectric coefficients. From Fig. 4, when the static and dynamic flexoelectric coefficients have opposite signs, the flexoelectric effect always reduces the natural frequencies. Conversely, when the case of the same sign, the flexoelectric effect increases the natural frequencies. In this case study, it is noted that the change in natural frequencies can reach nearly 28% due to the choice of the relative sign and values of the flexoelectric coefficients.

### Effect of the surface effects

**Fig. 5:**
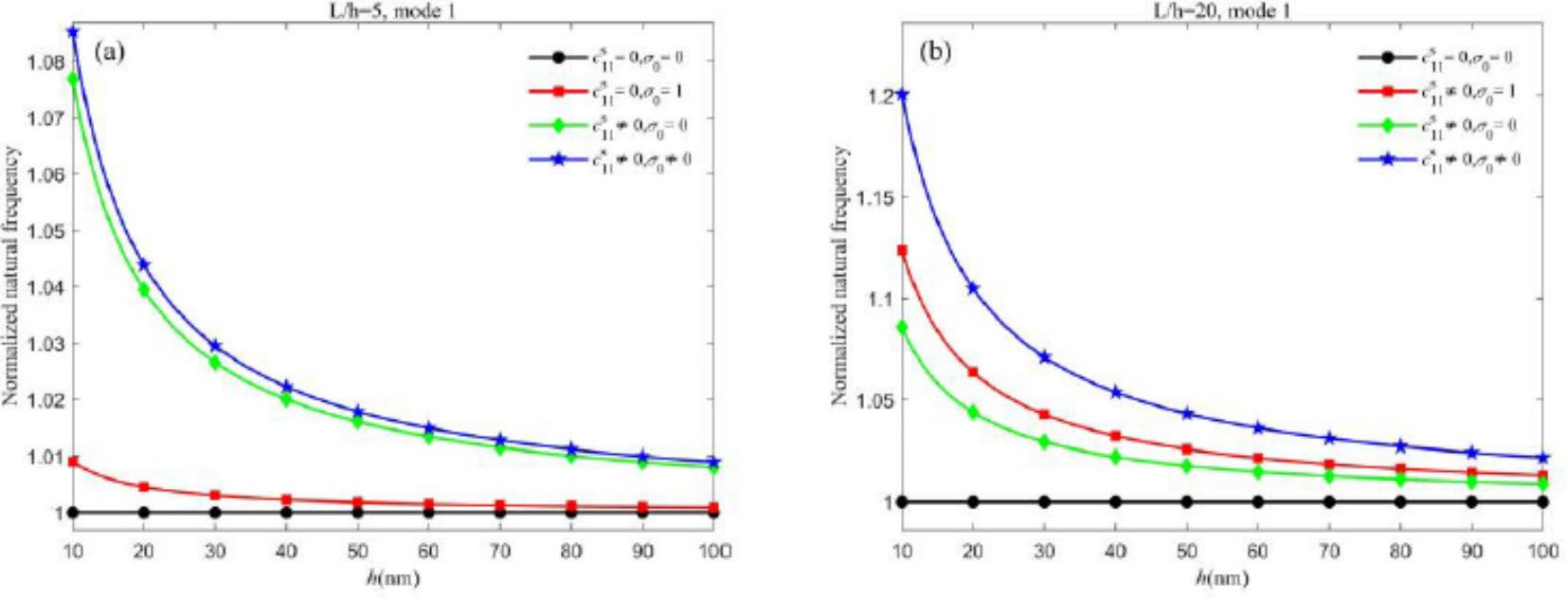
Curves of normalized natural frequency changes with thickness *h* for different surface effects (a) Timoshenko beam (b) Euler-Bernoulli beam.

When the flexoelectric effect is not considered, the surface effect on the natural frequencies is examined. Figure 5(a) and 5(b) show the normalized natural frequencies of the Timoshenko nanobeam and Euler-Bernoulli nanobeam with different $c_{{11}}^{s}$ and ${\sigma _0}$, respectively. It can be seen that positive surface residual stress ${\sigma _0}$ increases the natural frequencies, while negative surface residual stress ${\sigma _0}$ reduces the natural frequencies. The surface elasticity $c_{{11}}^{s}$ can also increase the natural frequencies, which is due to the hardening of the nanobeam’s surface. From Fig. 5(a) and 5(b), we find that the effect of surface elasticity $c_{{11}}^{s}$ is more significant than that of surface residual stress ${\sigma _0}$ on the natural frequencies. By comparing Fig. 5(a) and 5(b), it can be found that the natural frequency of the Timoshenko nanobeam differs from that of the Euler-Bernoulli nanobeam under the same conditions. Specifically, when the thickness of the nanobeam is small, the natural frequency of the Timoshenko nanobeam is greater than that of the Euler-Bernoulli nanobeam. This is because the impact of shear strain gradient on the natural frequencies is prominent for thinner nanobeams. The difference in the natural frequency between the two types of nanobeams decreases as the thickness increases.

**Fig. 6:**
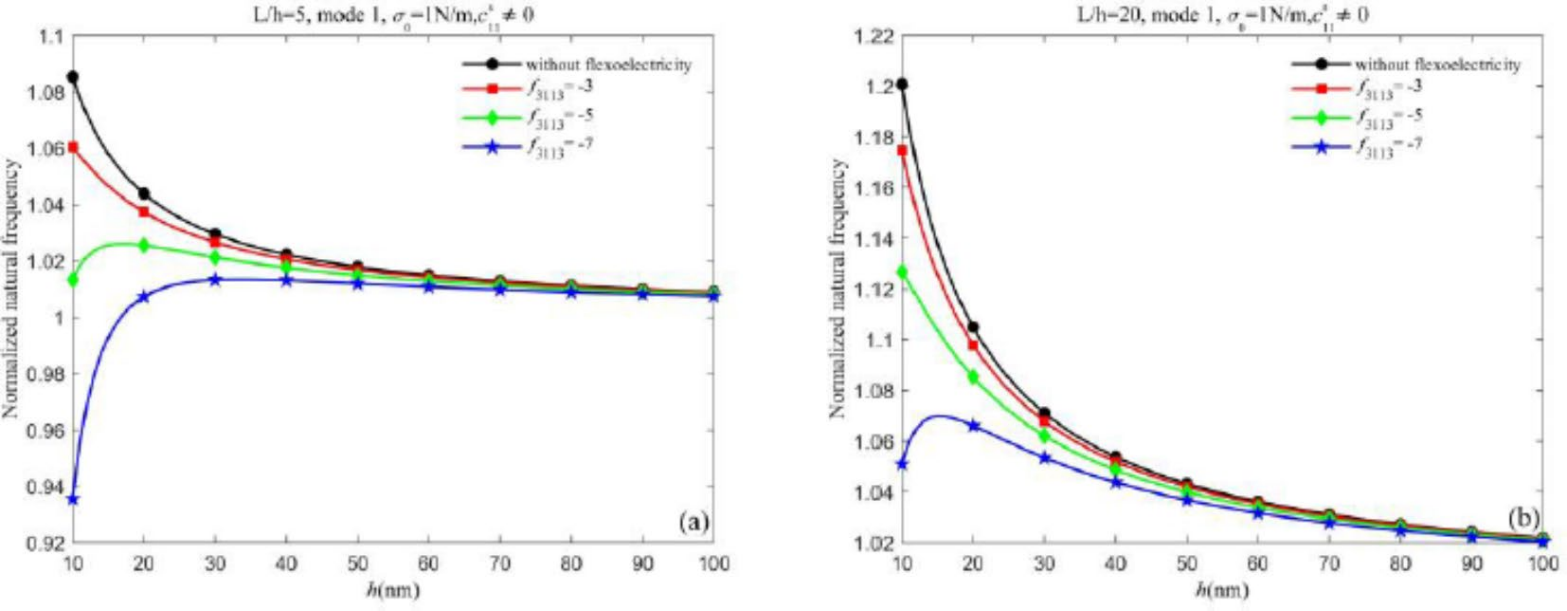
The influence of flexoelectric effect and surface effect on normalized natural frequency for different static flexoelectric coefficients (a) Timoshenko beam (b) Euler-Bernoulli beam.

Furthermore, when the static flexoelectric effect is considered, Fig. 6(a) and (b) display the normalized natural frequency with thickness *h* for both the piezoelectric nanobeams under different static flexoelectric coefficients, respectively. As shown in Fig. 6, the surface and static flexoelectric effects have a competitive impact on the natural frequencies of nanobeams. When the static flexoelectric coefficient is small, the natural frequency is mainly influenced by the surface effect and decreases with the increase of the thickness. However, as the static flexoelectric coefficients continue to increase, the contribution of the static flexoelectric effect gradually increases even becomes the primary factor.

**Fig. 7:**
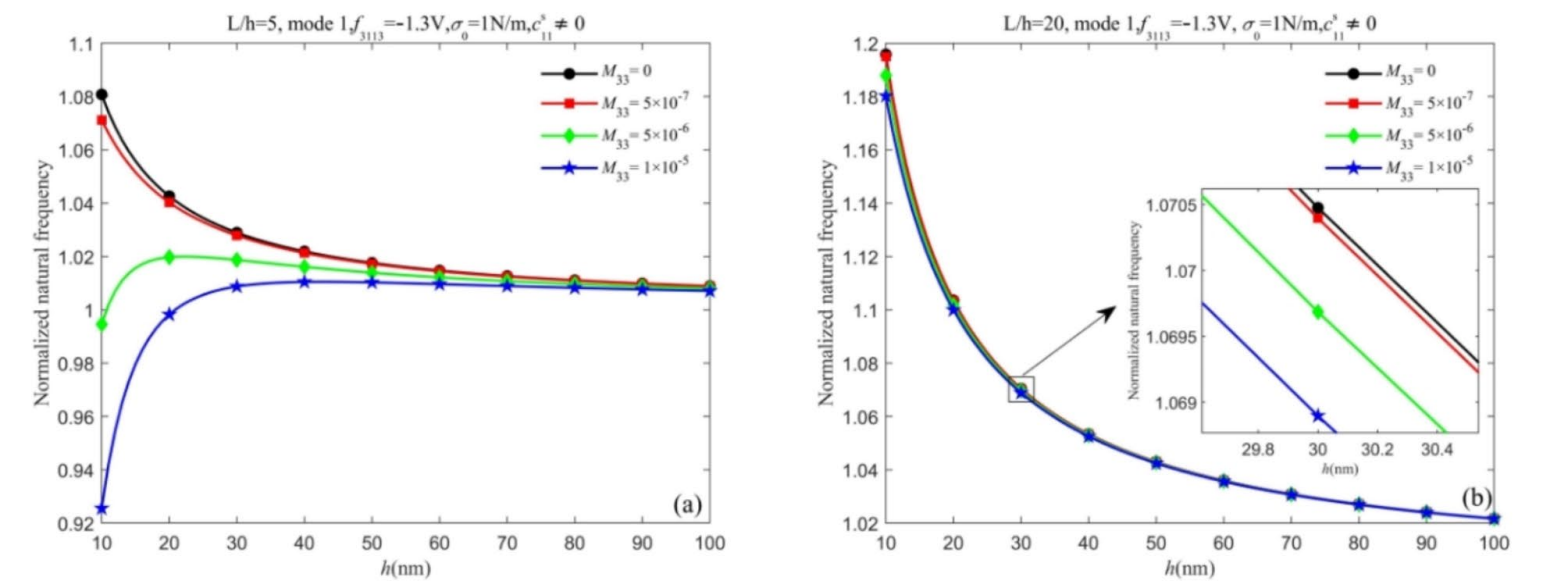
Curves of normalized natural frequency of nanobeams change with thickness *h* for different dynamic flexoelectric coefficients (a) Timoshenko beam (b) Euler-Bernoulli beam.

From the conclusions drawn in Fig. 6, the proportion of the static flexoelectric effect and surface effect to the total effect needs further discussion. Thus, in Fig. 7, we consider the dynamic flexoelectric effect and discuss the proportion of the three effects on the natural frequencies with different dynamic flexoelectric coefficients. From Figs. 7 and 8, it is observed that the natural frequencies predicted by the Timoshenko beam model and Euler-Bernoulli beam model are less than those when the dynamic flexoelectric effect is not considered. The dynamic flexoelectric effect dominates when the dynamic flexoelectric coefficient is large enough, the normalized natural frequency is less than 1 and increases as the thickness increases. However, the normalized natural frequency reaches a maximum greater than 1 due to the surface effect and then gradually drops down with the increase in thickness. The explanation for this phenomenon is that the dynamic flexoelectric effect has a stronger size dependence compared to the surface effect. It is worth noting that the normalized natural frequency remains greater than 1 due to the surface effect, which cannot be completely canceled out.

## Conclusions

In this work, we have studied the dynamic flexoelectric effect on the free vibration of flexoelectric nanobeams with the surface effect. The main contributions of this paper include the derivation of the governing and frequency equations for flexoelectric nanobeams with dynamic flexoelectric and surface effects. The main conclusions are as follows:

1. The dynamic flexoelectric effect significantly affects the natural frequency of Timoshenko beam model, while the effect on the natural frequency of Euler-Bernoulli beam model is subtle and even negligible. For nanobeams with higher vibration modes, the influence of dynamic flexoelectric effect on the natural frequency is more influential.
2. When the signs of the static and dynamic flexoelectric coefficients are opposite, the natural frequencies decrease as the flexoelectric coefficients increase, and when the case of the same sign, the trend is reversed.
3. In the presence of the surface effect, positive surface residual stress increases the natural frequencies, while negative surface residual stress reduces the natural frequencies. The surface effect is more influential on the Timoshenko beam model.
4. When the flexoelectric coefficient is small or the beam is sufficiently thick, the natural frequency is mainly influenced by the surface effect. Nevertheless, the contribution of the flexoelectric effect gradually increases even becoming the primary factor as the flexoelectric coefficients increase.
5. The surface and flexoelectric effects have a competitive impact on the natural frequencies and the flexoelectric effect has a stronger size dependence than the surface effect.

These results are helpful for the design of intelligent nanodevices. It should be noted that the limitation of this paper is the use of classical elasticity theory, which may not be sufficiently accurate for nanoscale structures^27^. Analyzing the impact of the three effects based on modified strain gradient theory is planned for future work^15–17^.

## Data Availability

All data generated or analysed during this study are included in this published article.
